# Fast Diffusion Tensor Magnetic Resonance Imaging of the Mouse Brain at Ultrahigh-Field: Aiming at Cohort Studies

**DOI:** 10.1371/journal.pone.0053389

**Published:** 2012-12-28

**Authors:** Hans-Peter Müller, Ina Vernikouskaya, Albert C. Ludolph, Jan Kassubek, Volker Rasche

**Affiliations:** 1 Department of Neurology, University of Ulm, Ulm, Germany; 2 Experimental Cardiovascular Imaging, Core Facility Small Animal MRI, University of Ulm, Ulm, Germany; University of Maryland, College Park, United States of America

## Abstract

**Introduction:**

*In-vivo* high resolution diffusion tensor imaging (DTI) of the mouse brain is often limited by the low signal to noise ratio (SNR) resulting from the required small voxel sizes. Recently, cryogenically cooled resonators (CCR) have demonstrated significant increase of the effective SNR. It is the objective of this study to enable fast DTI of the mouse brain. In this context, CCRs appear attractive for SNR improvement.

**Methods:**

Three mice underwent a DTI examination at 156^2^×250 µm^3^ spatial resolution with a CCR at ultrahigh field (11.7T). Diffusion images were acquired along 30 gradient directions plus 5 references without diffusion encoding, resulting in a total acquisition time of 35 minutes. For comparison, mice additionally underwent a standardized 110 minutes acquisition protocol published earlier. Fractional anisotropy (FA) and fiber tracking (FT) results including quantitative tractwise fractional anisotropy statistics (TFAS) were qualitatively and quantitatively compared.

**Results:**

Qualitative and quantitative assessment of the calculated fractional anisotropy maps and fibre tracking results showed coinciding outcome comparing 35 minute scans to the standardized 110 minute scan. Coefficients of variation for ROI-based FA-comparison as well as for TFAS revealed comparable results for the different scanning protocols.

**Conclusion:**

Mouse DTI at 11.7 T was performed with an acquisition time of approximately 30 minutes, which is considered feasible for cohort studies. The rapid acquisition protocol reveals reliable and reproducible FA-values and FT reconstructions, thus allowing an experimental setup for *in-vivo* large scale whole brain murine DTI cohort studies.

## Introduction

In the last decade, diffusion tensor imaging (DTI) [1], [2] has evolved as an increasingly important tool for studying the anatomy of the mouse brain *in-vitro* [3]–[6] as well as *in-vivo* [7]–[13]. However, to establish DTI as a broad pre-clinical research tool to study white matter alterations in animal models, further optimization of the DTI protocols especially regarding acceptable data acquisition times appears mandatory.

As an *in-vivo* approach, DTI is able to map the brain fiber directions and to render its 3D architecture, thus reconstructing axonal tracts and especially tracking the white matter pathways of the human brain [3]. DTI characterizes the diffusion of water molecules in tissue by using motion-probing spatial encoding [1]: For each voxel of the image, the diffusion tensor describes the magnitude and directionality of the water movement (anisotropy). Tractography algorithms use this information to track the neural pathways based on the assumption that the dominant direction of water motion – the main axis of the diffusion tensor – aligns with the fibers' orientations in an imaged voxel [3]. This allows for anatomical connectivity studies providing insights to brain function and morphology under physiological conditions or to dysfunction under pathological circumstances. DTI studies of the rodent brain in high-field animal scanners with 7.0 up to 11.7T [7]–[13] require long scan times to ensure appropriate signal-to-noise ratio (SNR) at sufficient spatial resolution (see Supplementary Table S1). Current in-plane resolutions for in-vivo experiments were ranging between 117 µm and 160 µm with a slice thickness between 375 µm and 1 mm. Total acquisition times for a slice thickness of 500 µm and lower were reached by acquisition times of 1–2 hours [11], [13]. High-resolution experiments have been conducted on in-vitro brain tissue even to conduct fiber tracking (FT) and consequent tract based spatial statistics (TBSS) [6]. Obviously, the in-vitro scans cannot be applied to longitudinal studies. Furthermore, the main advantage of DTI enabling a safe and non-invasive approach for longitudinally investigating normal development, aging, disease progression, etc. cannot be ensured by the in-vitro approach.

In-vivo experiments have been reported with in-plane resolution down to 156 µm×156 µm (matrix size 128×92) with an axial slice thickness of 375 µm and 500 µm [11], [13]. Although the spatial resolution revealed acceptable FT information, the corresponding long scanning time of about 90 minutes limits the application of the proposed protocol for large cohort studies.

The aim of the study was to investigate the feasibility of *in vivo* microstructural analysis of the mouse brain including fiber tracking by applying a rapid data acquisition protocol providing sufficient spatial resolution in short scan times. Reduction of scanning time without compromising spatial resolution is obtained by application of a cryogenic coil at 11.7T field strength. Cryogenically cooled resonators (CCR) have been shown to significantly increase the effective SNR (factor between 2.4 and 2.5 in structural MRI scans) at 4.7T [14] as well as at 9.4 T [15] and are supposed to provide an SNR gain of at least a factor of 2 at 11.7T systems. The SNR gain is supposed to generate high fidelity DTI data with high in-plane resolution and thin slices in order to get close to isotropic voxel resolution, which improves isotropic 3D DTI fiber reconstruction. The aim is to keep the overall scanning time at approximately 30 minutes in order to enable the application of the scan protocol to *in-vivo* cohort studies. Metrics applied for the evaluation of the quality of the resulting fiber data include the quality of the FA-maps and the quality of fiber reconstruction, which is tested quantitatively by tractwise fractional anisotropy statistics (TFAS) [16].

## Results

**Figure 1:**
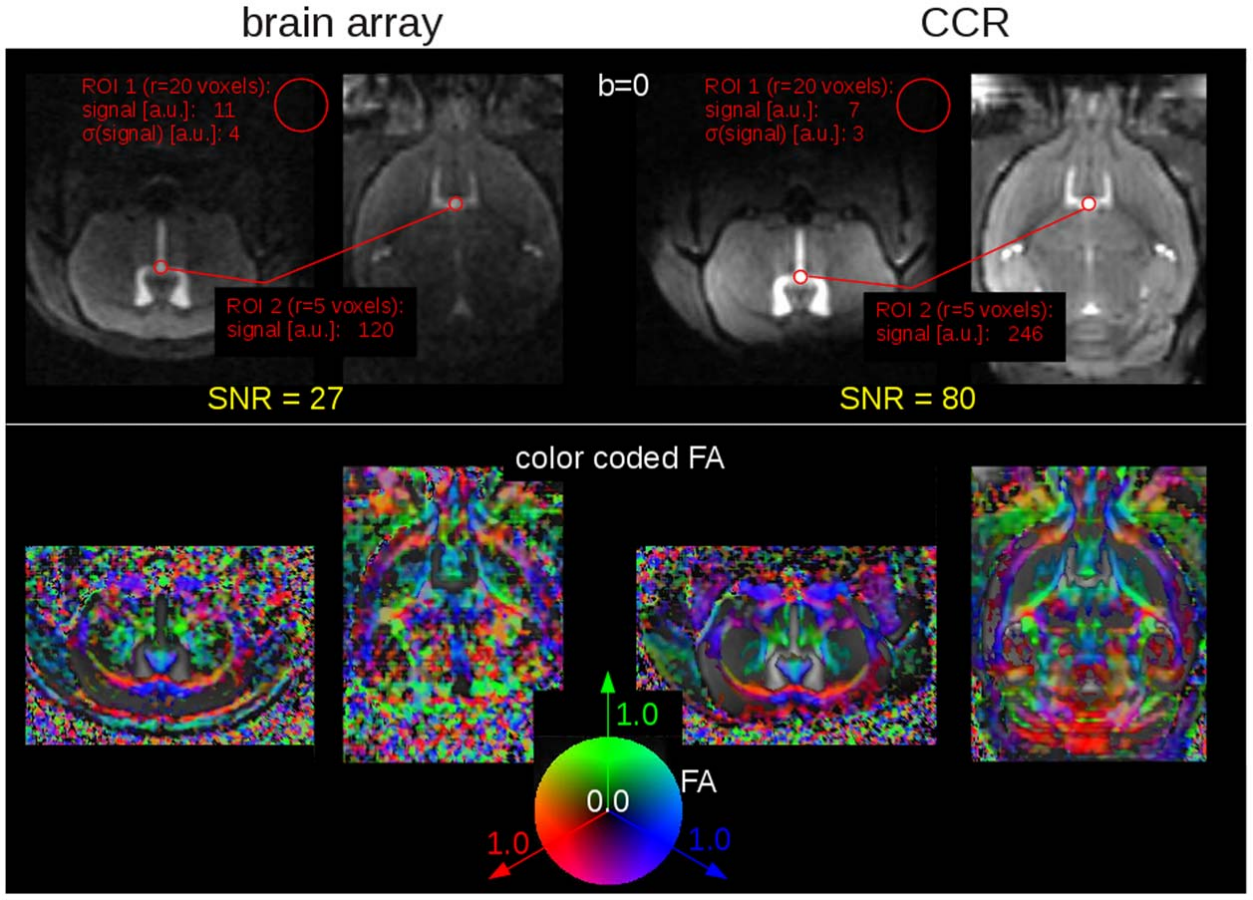
Brain array coil (left) vs. cryogenic cooled resonator (CCR – right). **Upper panel:** (b = 0) anatomical images (axial slice and coronal reconstruction) used for signal-to-noise ratio (SNR) estimation: ROI 1 (r = 20 voxels) is located in a region without signal in order to estimate the noise, ROI 2 (r = 5 mm) is located in the ventricles in order to estimate the signal intensity in a region with high (b = 0)-signal. **Lower panel:** Directional encoded color maps of FA (axial slice and coronal reconstruction) - directional information was incorporated by color coding the scalar FA map with the red, green and blue colors to label the left-right, ventral-dorsal, and caudal-rostral directions, respectively.

The direct comparison of the image quality and resulting FA maps acquired with the rapid DTI protocol with a dedicated four-element brain coil and a two-element cryogenic brain coil is shown in Figure 1. The gain in SNR and the respective improvement in the FA maps is obvious. However, since the CCR is a transmit/receive coil, the SNR gain is not homogenous over the whole brain. Highest gain is achieved in an about 3 mm region in the midbrain in which the RF transmit power was optimized. In the anterior and posterior sections, the SNR gain is decreasing due to the reduced B1-fields causing imperfect excitation and refocusing. Average gain in SNR over the brain is about a factor of 2.3 with peak values close to 3 as shown in Figure 1.

**Figure 2:**
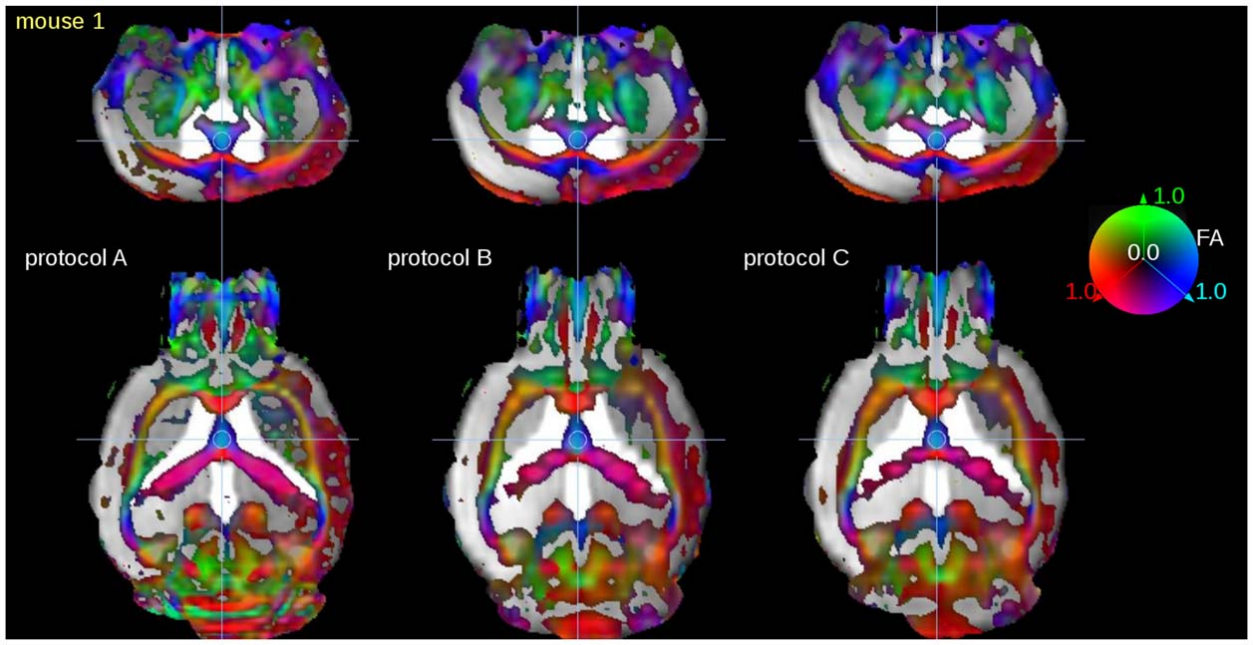
Directional encoded color maps of FA overlaid into (b = 0) anatomical images: comparison of FA quality for scanning protocols (SPs) A (35 minutes, 1 average), B (110 minutes, 6 averages), and C (18 minutes, 1 average). Coronal slices (**upper panel**) show only slight visual differences. The lower slice thickness of SP A (compared to SPs B and C) is visible in the reconstructed axial slices (**lower panel**). Directional information was incorporated by color coding the scalar FA map with the red, green and blue colors to label the left-right, ventral-dorsal, and caudal-rostral directions, respectively. FA-display threshold was 0.2.

The visual comparison of color coded FA representations obtained from the three different scanning protocols (SPs) with the cryogenic coil is given in Figure 2 exemplarily for mouse 1. Reconstruction of the coronal slices clearly shows the improved axial resolution (250 µm in SP A compared to 500 µm in SPs B and C).

**Figure 3:**
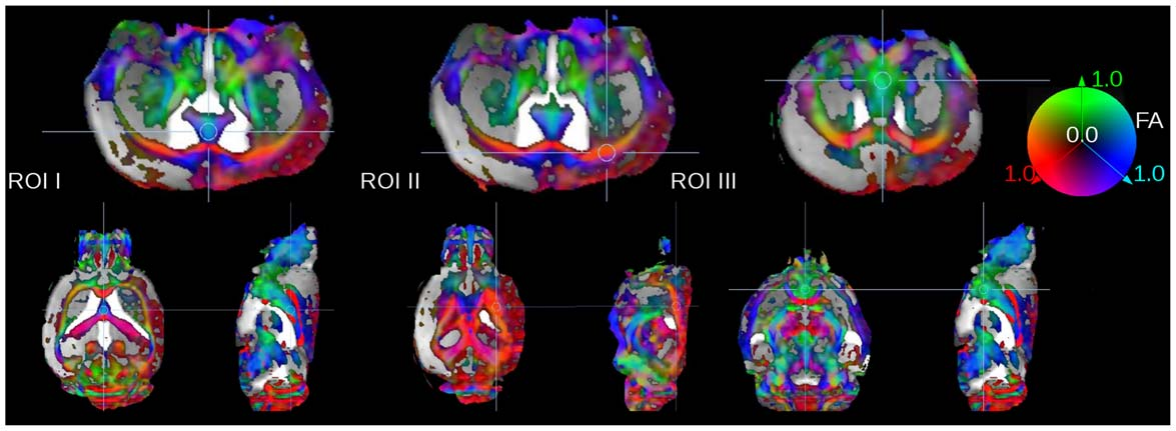
Location of ROIs for FA-value calculation in the septohippocampal nucleus (ROI I), in the corpus callosum (ROI II), and in the medial septal nucleus (ROI III). Directional information was incorporated by color coding the scalar FA map with the red, green and blue colors to label the left-right, ventral-dorsal, and caudal-rostral directions, respectively. FA-display threshold was 0.2.

**Figure 4:**
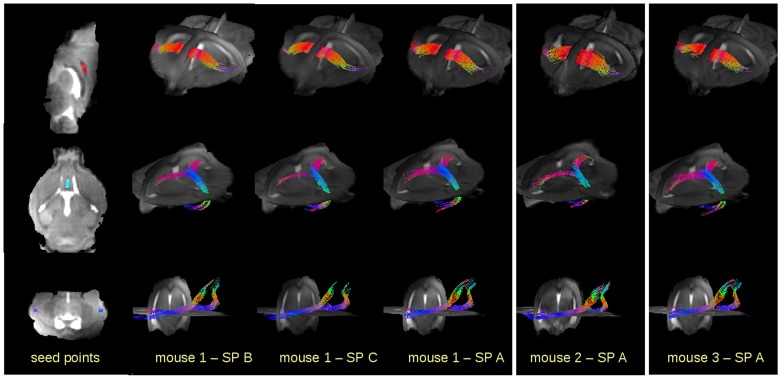
FT results for seed points in the genu and along the corpus callosum, near the lateral septal nucleus, and in the olfactory path. **Left column:** Location of the seed points. **Columns 2–4:** FT for mouse 1 with different scanning protocols (SP). **Columns 5–6:** FT for mice 2 and 3, respectively, with SP A (35 minute scan).

**Figure 5:**
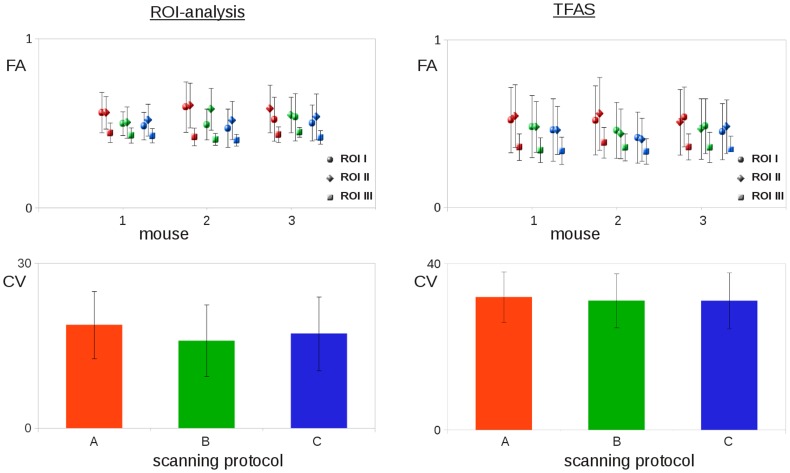
Statistical analysis. **Left panel** – ROI analysis: FA-values (standard deviation as error bars) for different mice with different scanning protocols (SP) (c*olors red, green, blue represent scanning protocols A,B,C, respectively)* and coefficients of variance (CV) averaged for 3 different ROIs in 3 different mice for the 3 scanning protocols. **Right panel** – TFAS: FA-values (standard deviation as error bars) for different mice with different scanning protocols (SP) (c*olors red, green, blue represent scanning protocols A,B,C, respectively)* and coefficients of variance (CV) averaged for 3 different TFAS in 3 different mice for the 3 scanning protocols.

Quantitative comparison was performed by ROI analysis (Figure 3) as well as by TFAS (Figure 4). The comparison of FA values in selected ROIs (Figure 3) revealed an inter-subject variability for the different ROIs (I–III) for the different mice below 0.10, which is a similar range as reported by [11] (Figure 5, upper panel, left). TFAS results (Figure 4) also revealed only slight differences between the different mice (Figure 5, upper panel, right). For ROI analysis, lowest coefficient of variation (CV) was detected for SP B corresponding to the 110 minutes scan. A slight decrease in FA-values and increased CV was observed by comparing 1 signal average to 6 signal averages (SPs C and B) caused by lower SNR. Highest FA values and highest CV were observed for the optimized rapid scanning protocol SP A. Although FA values were higher for SP A in TFAS, CV revealed almost identical CV for all SPs.

## Discussion

In this study, the feasibility of *in-vivo* fiber tracking and microstructural analysis of the mouse brain by use of a rapid data acquisition protocol of 35 minutes with a cryogenic coil has been investigated. Sufficient spatial resolution was provided, and a post-processing protocol equivalent to human group studies could be applied. Voxel sizes of 150 µm provide diffusion information in the dimension of axon bundles. As FA is a measure of the diffusion directionality, the voxel size is supposed to influence the FA values. Since with larger voxel size (or slice thickness) more axons are averaged, a likely decrease in FA values due to lower directionality is observed (Figure 5, compare FA values (ROI analysis as well as TFAS). This does not necessarily hold in areas with coherent white matter tracts because axons are well packed with high directionality in those coherent tracts. Here, a possible explanation of FA drop in large voxels is the higher likelihood of partial volume effect from neighboring gray matter or CSF which can easily decrease the overall FA. This was confirmed in the comparison of SP A (156^2^×250 µm^3^) with SPs B and C, respectively (156^2^×500 µm^3^) (Figures 2, 5).

High voxel resolution and low slice thickness in the FA-maps enabled high quality FT in all data sets. ROI analysis as well as TFAS analysis results showed FA variability in the range of inter-subject variability. The FA threshold in FT of 0.2 [17] which has already been proven to be a good choice for human DTI could also be used as a valid threshold for murine DTI. Further improvement of the data quality could be achieved by optimization of the gradient sequence by a broader range of b-values. This should be topic of further studies.

The cryogenic coil revealed high quality signals (SNR improvement by a factor up to 3) which in this study allowed for the optimization of a DTI protocol with a total acquisition time of approximately 30 minutes. DTI scans with only a single signal average provide the opportunity to accurate calculate diffusion tensors and the consecutive FA-maps resulting in reliable FA-values and FT reconstructions. In order to gain high resolution FT, small voxel volumes are essential, i.e. small voxel size in-plane combined with thin slice thickness. The high slice resolution of 250 µm allows for FT almost on an iso-grid.

The experimental setup of this study allows for *in-vivo* large scale whole brain murine DTI cohort studies. Thus, this study claims to be a step forward to establish murine DTI in pre-clinical neuroimaging.

## Materials and Methods

### 1. Animals

All experiments were performed in accordance with German animal protection laws and had been approved by the national animal board (TVA 1001, “Etablierung Kleintierbildgebung an 3T und 11.7T”, Regierungspräsidium Baden-Württemberg, Tübingen, Germany). Three adult wild type mice (C57/B6, 12 months old) underwent the whole-brain DTI-MRI protocol. All data acquisition was carried out under isoflurane anesthesia (3% for induction and 1.5% for maintenance).

The animals were placed in a stereotactic head support (Bruker Biospin, Ettlingen, Germany) to immobilize the head. Body temperature was controlled by an integrated water based heating device. The actual body temperature of the mouse was monitored by a rectal temperature probe and respiration was monitored by a respiratory pillow positioned under the abdomen of the mouse. The breathing frequency was maintained at 75–80 cycles per minute. The mice rapidly recovered (<5 min) after the termination of anesthesia at the end of the MRI procedure.

### 2. Data acquisition

Imaging was performed with an 11.7T small bore animal scanner (Biospec 117/16, Bruker, Ettlingen, Germany). A two-element transmit/receive ^1^H mouse cryogenic surface coil (CryoProbe, Bruker BioSpin) was used for data acquisition. For direct SNR comparison, in two mice data were additionally acquired with SP A by a dedicated four-element receive only surface coil. Imaging parameters of the optimized rapid diffusion prepared spin echo EPI imaging protocol were as: TE/TR 50.5 ms/15000 ms, matrix 128×96, in-plane resolution 156 µm×156 µm, 60 axial slices with a slice thickness of 250 µm. Thirty diffusion directions with b = 1000 s/mm^2^ and 5 unweighted b = 0 volumes (standard gradient scheme as provided by the Bruker software), 1 signal average, were acquired, resulting in a total acquisition time of 35 minutes (SP A). For comparison, an additional acquisition according to the proposed protocol of Harsan et al. [11] was performed with acquisition parameters as standard protocol: TE/TR 20.5 ms/7750 ms, matrix 128×96, in-plane resolution 156 µm×156 µm, 30 axial slices with a slice thickness of 500 µm, diffusion scheme identical as before. Six signal averages resulted in a total acquisition time of 110 minutes (SP B). SP C was identical to SP B but included one signal average, resulting in 18 minutes scan time. No respiratory or cardiac synchronization was used.

### 3. Data analysis/processing

Signal-to-noise ratios (SNR) were calculated according to:
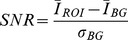
with 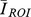 being the mean value of a manually drawn region of interest, 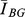 the mean background signal, and 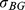 the standard deviation of the background signal.

Data preprocessing was performed with the *Tensor Imaging and Fiber Tracking* (TIFT) software package [18] which has been successfully applied to human DTI group studies (e.g. [19]). After transformation of the recorded data into a 50 µm isogrid (nearest neighbor interpolation), fractional anisotropy (FA) maps were calculated. FA values were displayed by directional encoded color maps of FA overlaid into (b = 0) anatomical images: directional information was incorporated by color coding the scalar FA map with the red, green and blue colors to signify the left-right, ventral-dorsal, and caudal-rostral directions, respectively (Figure 2). FA-display threshold was 0.2. For a better comparability, data sets were aligned along the corpus callosum using affine transformations. No co-registration by non-affine transformations was used.

ROI analysis of FA-values was performed in 3 spheric ROIs with a radius of 10 voxels (500 µm) (Figure 3). The location of the ROIs was set manually in each mouse, adapted to the different morphology of the mouse brains which had not been co-registered. ROIs were located in the septohippocampal nucleus (ROI I), in the coprus callosum (ROI II), and in the medial septal nucleus (ROI III).

FT was performed for identification of some major pathways with seed points located at the genu and along the corpus callosum, near the lateral septal nucleus, and in the olfactory tract (Figure 4). The location of the seed points for FT (Figure 4, left panel) was set manually, adapted to the different morphology of the mouse brains. TFAS was performed in the respective Fts. FT was performed by deterministic streamline tracking technique [16], [20] using an FA threshold of 0.2 [17]. In order to obtain quantitative assessment to the tractography results, tractwise fractional anisotropy statistics (TFAS) [16] was applied for comparison of FT results of the different DTI data sets.

In order to obtain an estimation of the signal-to-noise (SNR), the CV was used. The CV is defined as the ratio of the measurements standard deviation divided by the mean and multiplied by 100. It is an estimate of measurement variance expressed as relative percentage, regardless of the absolute measurement value. In previous studies on DTI test–retest reliability, the CV is the most commonly reported statistical measure [21] CVs of FA data were computed in different regions-of-interest (ROI) as well as for the results of the TFAS.

## Supporting Information

Table S1 Overview of recently used scanning parameters in murine DTI.
(DOC)
