# Supplementary material for: Fast Diffusion Tensor Magnetic Resonance Imaging of the Mouse Brain at Ultrahigh-Field: Aiming at Cohort Studies
Source: PLoS One. 2012 Dec 28;7(12):e53389. doi: 10.1371/journal.pone.0053389 (PMC3532447; doi:10.1371/journal.pone.0053389)
Supplement: Table S1 — Overview of recently used scanning parameters in murine DTI. (DOC) [file pone.0053389.s001.doc]

*Supplementary Table 1:*

| **reference** | **in-plane (µm)** | **slice (µm)** | **field (T)** | **in vivo** | **scan time** | **isoflurane (%)** |
| --- | --- | --- | --- | --- | --- | --- |
| *Boretius et al., 2007 [7]* | 150 x 150 | 1000 | 7.0 | yes | 23 min | 1.5 - 2 |
| Chuang et al., 2011 [3] | 80-125 iso | 80-125 | 11.7 | no | 24 h | - |
| Guilfoyle et al., 2011 [8] | 117 x 117 | 1000 | 7.0 | yes | 30 min | 1 |
| Gutman et al., 2012 [4] | 161 x 161 | 161 | 9.4 | no | 60 h | - |
| Jiang et al., 2011 [9] | 43 x 43 | 43 | 9.4 | no | 28 h | ? |
| Kumar et al., 2012 [10] | 156 x 156 | 800 | 9.4 | yes | 2 h | 3 |
| Pathak et al., 2011 [5] | 94 x 97 | 600 | 9.4 | no | ? | - |
| Ruest et al., 2011 [6] | 130 x 130 | 130 | 7 | no | 14 h | - |
| Harsan et al. 2010 [11] | 156 x 156 | 500 | 9.4 | yes | 90 min | 1.5 |
| Aggarwal et al., 2009 [12] | 117 x 125 | 375 | 9.4 | yes | 2.2 h | ? |
